# Safety of subconjunctival allogeneic platelet-rich plasma on normal canine eyes

**DOI:** 10.3389/fvets.2026.1870669

**Published:** 2026-07-20

**Authors:** Amanda B. Davis, Mia L. Stewart, Abby A. Hickox

**Affiliations:** Department of Veterinary Clinical Sciences, College of Veterinary Medicine, The Ohio State University, Columbus, OH, United States

**Keywords:** allogeneic platelet-rich plasma, canine, platelet-rich plasma injection, subconjunctival injection, veterinary ophthalmology

## Abstract

**Purpose:**

To determine the safety and tolerance of an allogeneic, pooled freeze-dried platelet-rich plasma (PRP; VetStem PrecisePRP™ Canine) product administered via subconjunctival injection two weeks apart in clinically normal canine eyes.

**Methods:**

Allogeneic PRP was reconstituted with sterile water. With topical anesthesia and gentle manual restraint of each dog, 0.2 mL (500 × 10^6^ platelets/mL) of the reconstituted preparation was injected under the dorsal bulbar conjunctiva of the right eye. The procedure was repeated with 0.2 mL of sterile saline (control) under the dorsal bulbar conjunctiva of the left eye. A complete blood count, chemistry profile, and comprehensive physical and ophthalmic examinations were conducted on all dogs at multiple time points to monitor for potential adverse reactions.

**Results:**

No significant adverse effects associated with physical examination, hematologic or ocular parameters were noted at any time point during the study. All allogeneic PRP treated eyes developed a mild to moderate conjunctival hyperemia and chemosis that self-resolved within 48–72 h. Additionally, one eye developed a mild periocular hyperemia that self-resolved within 72 h. No adverse reaction was noted in any saline treated eye.

**Conclusion:**

Allogeneic PRP administered via subconjunctival injection in normal canine eyes appears to be safe and well-tolerated with only transient conjunctival hyperemia and chemosis noted. This study allows for further investigation into the clinical efficacy of subconjunctival allogeneic PRP for canine ocular surface inflammatory disorders.

## Introduction

Ocular surface diseases, such as keratoconjunctivitis sicca, are frequently encountered in veterinary patients and may leave the ocular surface particularly susceptible to chronic inflammation, immune activation/dysregulation, and tissue damage (1, 2). Most therapeutic interventions for these inflammatory disorders are palliative in nature; therefore, strategies aimed at modulating and repairing the local immune environment may offer significant benefit for these common and occasionally unmanageable conditions.

In recent years, platelet-rich plasma (PRP) has emerged as a promising therapeutic option in both human and veterinary medicine for the management of a broad range of conditions. PRP is the plasma fraction of whole blood with a platelet concentration above physiologic baseline (3). It contains serum, platelets, leukocytes, and an array of growth factors, including vascular endothelial growth factor (VEGF), platelet-derived growth factor (PDGF), transforming growth factor-beta (TGF-*β*), epidermal growth factor (EGF), insulin-like growth factor (IGF), and fibroblast growth factor (FGF) (4, 5). These bioactive molecules promote collagen synthesis, angiogenesis, cytokine alteration, epithelial differentiation, endothelial cell proliferation, and other reparative processes (4–6).

Due to its regenerative and anti-inflammatory properties, PRP has been heavily utilized in the treatment of musculoskeletal disorders; however, PRP treatment of the ocular surface has been and continues to be heavily investigated. PRP can be administered in many ophthalmic forms such as topical drops, subconjunctival injections, and clot application which has a particular focus in the treatment of corneal and ocular surface disorders (4, 7). Preliminary evidence supports the use of PRP in the treatment of dry eye disease, corneal chemical and thermal injuries, epithelial defects, keratitis, and corneal ulceration in both veterinary and human patients (4, 5, 8, 9). A recent systematic review of PRP within the veterinary ophthalmic literature notes that PRP is associated with a reduction of inflammation in corneal healing and animal ocular surface disease (OSD) scores; however, there are limitations in study design and variability in PRP preparation (10).

PRP can be derived autologously (from the patient) or allogeneically (from a donor of the same species). Autologous PRP has been widely studied and shown to be safe, inexpensive, and effective in regenerative applications (4, 8, 11). However, significant variability exists in autologous PRP preparation methods, including differences in anticoagulants, centrifugation practices, and collection volumes. These variables affect platelet and leukocyte concentrations and consequently alter the therapeutic potential of the final PRP product (4, 5, 12). Even commercially available PRP preparation systems lack standardization across manufacturers (13). Additionally, intrinsic patient factors, such as hydration status, medication use, age, etc. and variation in protocol may further influence final platelet yield (4, 14). This lack of consistency is one of the primary limitations in understanding the efficacy of autologous PRP in both human and veterinary studies (5). Processing time also creates practical challenges as preparation of autologous PRP commonly requires up to one hour. This may be impractical in a high-volume clinical setting (11).

Allogeneic PRP, while less extensively researched, has demonstrated comparable safety and efficacy profiles in the management of musculoskeletal conditions (4, 5, 15–19). Allogeneic formulations offer advantages over autologous, including a consistent product with known platelet concentrations, improved quality control, reduction in patient stress and visit times, and the potential for higher and more standardized platelet and growth factor concentrations. Notably, one human study reported significantly higher platelet counts and at least two-fold greater growth factor levels in allogeneic PRP compared to autologous PRP (20).

An FDA-reviewed, leucoreduced, canine allogeneic PRP product (VetStem PrecisePRP™ Canine) is already available to licensed veterinarians and has undergone thorough safety and quality testing for intra-articular use (21). Donor dogs are screened according to criteria from the Food and Drug Administration CVM Guidance #254. The product is shipped in freeze-dried vials, allowing convenient storage and reconstitution. An allogeneic PRP product offers the advantage of a standard composition and consistent quality of platelets to every patient, which allows wider access to this treatment option and improves its practicality in clinical use.

Numerous allogeneic PRP formulations have been evaluated as topical ophthalmic preparations in both human and veterinary models to assess their tolerability and efficacy in the management of various ocular surface disorders (22–28). Allogeneic PRP eye drops do not appear to induce adverse reactions due to their low immunogenicity. However, PRP treatment often requires frequent administration (4–6 times daily) over multiple weeks to achieve the desired effect (29, 30). At this frequency, a topical ophthalmic PRP preparation may be impractical for many veterinary clients, resulting in low compliance and inconsistent long-term results.

A subconjunctival route of PRP administration may alleviate treatment variability and reduce the degree of home care required. Additionally, a subconjunctival approach may enable more sustained delivery to the intended site and enhance regenerative potential through a depot-like effect that increases the local bioavailability of important growth factors (31, 32). Autologous subconjunctival PRP has already shown efficacy for a variety of ophthalmic disorders (7, 8, 33, 34). For the reasons discussed above, an allogeneic product may provide superior benefit; however, the safety of allogeneic PRP injected into the subconjunctival space has yet to be evaluated. The purpose of this study is to verify the safety and tolerance of an allogeneic PRP product (VetStem PrecisePRP™ Canine) injected into the subconjunctival space of normal canine patients. Due to its low immunogenicity and proven tolerance via other routes, we hypothesize that allogeneic PRP is safe with no significant adverse effects demonstrable when administered in this manner. Ultimately, if well-tolerated, this would translate into evaluating the efficacy of subconjunctival allogeneic PRP for the treatment of a variety of canine ocular surface conditions.

## Materials and methods

All care and use of research animals in this study was approved and monitored by the Ohio State University Institutional Animal Care and Use Committee (IACUC; protocol #2025A00000066).

All examination parameters were obtained and documented, and all injections were performed by a board-certified ophthalmologist (AD) or an ophthalmology resident (AH) under direct supervision of a board-certified ophthalmologist (AD). For this study, four healthy, 1-year-old intact female mixed large breed dogs were obtained and group-housed with two dogs to an enclosure in a climate-controlled environment. All dogs underwent comprehensive baseline physical examinations, including assessment of temperature, heart rate, respiratory rate and mucous membrane color with capillary refill time. Additionally, complete blood counts and serum chemistry profiles were obtained for all dogs. A complete ophthalmic examination was performed to include menace response, dazzle and pupillary light reflexes, Schirmer tear testing (Merck Animal Health, Madison, NJ, USA), fluorescein staining (JorVet, Loveland, CO, USA), tonometry (Tonometer, Dan Scott and Associates, Westerville, OH, USA), slit-lamp biomicroscopy (SL-17 Kowa Company Torrance, CA, USA) and indirect ophthalmoscopy. Dogs were excluded if they had evidence of ophthalmic or systemic disease.

Allogeneic, pooled freeze-dried platelet-rich plasma (PRP; VetStem PrecisePRP™ Canine) was reconstituted with 8 mL of sterile water as per label instructions. Approximately 0.1–0.2 mL of 0.5% proparacaine hydrochloride ophthalmic solution (Alcon Laboratories, Fort Worth, TX, USA) was administered topically to both eyes of each dog. Subsequently, 0.2 mL (500 × 10^6^ platelets/mL) of the reconstituted PRP preparation was drawn through a 22-gauge needle into a 1 mL syringe following gentle agitation. A 27-gauge needle was applied to the 1 mL syringe and, with gentle manual restraint of the dog, the platelets were injected just under the dorsal bulbar conjunctiva in the subconjunctival space of the right eye. The procedure was repeated for the left eye; however, 0.2 mL of sterile saline was administered into the dorsal bulbar subconjunctival space in place of PRP to serve as a control.

Full physical and ophthalmic examinations were performed in the same manner as baseline examinations at one hour and twenty-four hours post-injection. Standardized clinical photographs were obtained of all eyes. If any notable ocular adverse effects were still noted, slit-lamp biomicroscopic examination and photographs were repeated daily until clinical signs were resolved. Any adverse effects were graded according to the semi-quantitative preclinical ocular toxicology scoring criteria (SPOTS) generally accepted for standardizing slit lamp biomicroscopic findings in laboratory species (35). Daily observation was performed to assess general health (including temperature, heart rate and respiration), appetite, and attitude.

Fourteen days after the first injection, a comprehensive physical examination and ophthalmic examination, identical to the baseline examination, was repeated. A second injection was completed in the same manner as described above (0.2 mL PRP subconjunctival injection in the right eye and 0.2 mL sterile saline subconjunctival injection in the left eye) for each dog with gentle restraint and topical anesthesia. Full physical and ophthalmic examinations were performed at one hour and at minimum, one day post-injection. Standardized clinical photographs were obtained of all eyes. If any notable ocular adverse effects remained present, slit-lamp biomicroscopic examination and photographs were repeated daily until clinical signs were fully resolved. Comprehensive bloodwork (complete blood count and chemistry profile) was repeated one day after the second injection. Daily observation was performed to assess general health (including temperature, heart rate and respiration), appetite, and attitude until 7 days after the second injection, at which point the study was concluded.

## Results

**Figure 1:**
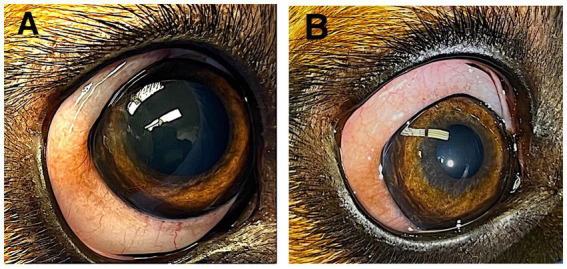
**(A,B)** Representative images of mild to moderate diffuse chemosis and hyperemia noted in all dogs one hour after the first PRP subconjunctival injection **(A)** and similarly, one hour after the second PRP subconjunctival injection performed 2 weeks later **(B)**.

**Figure 2:**
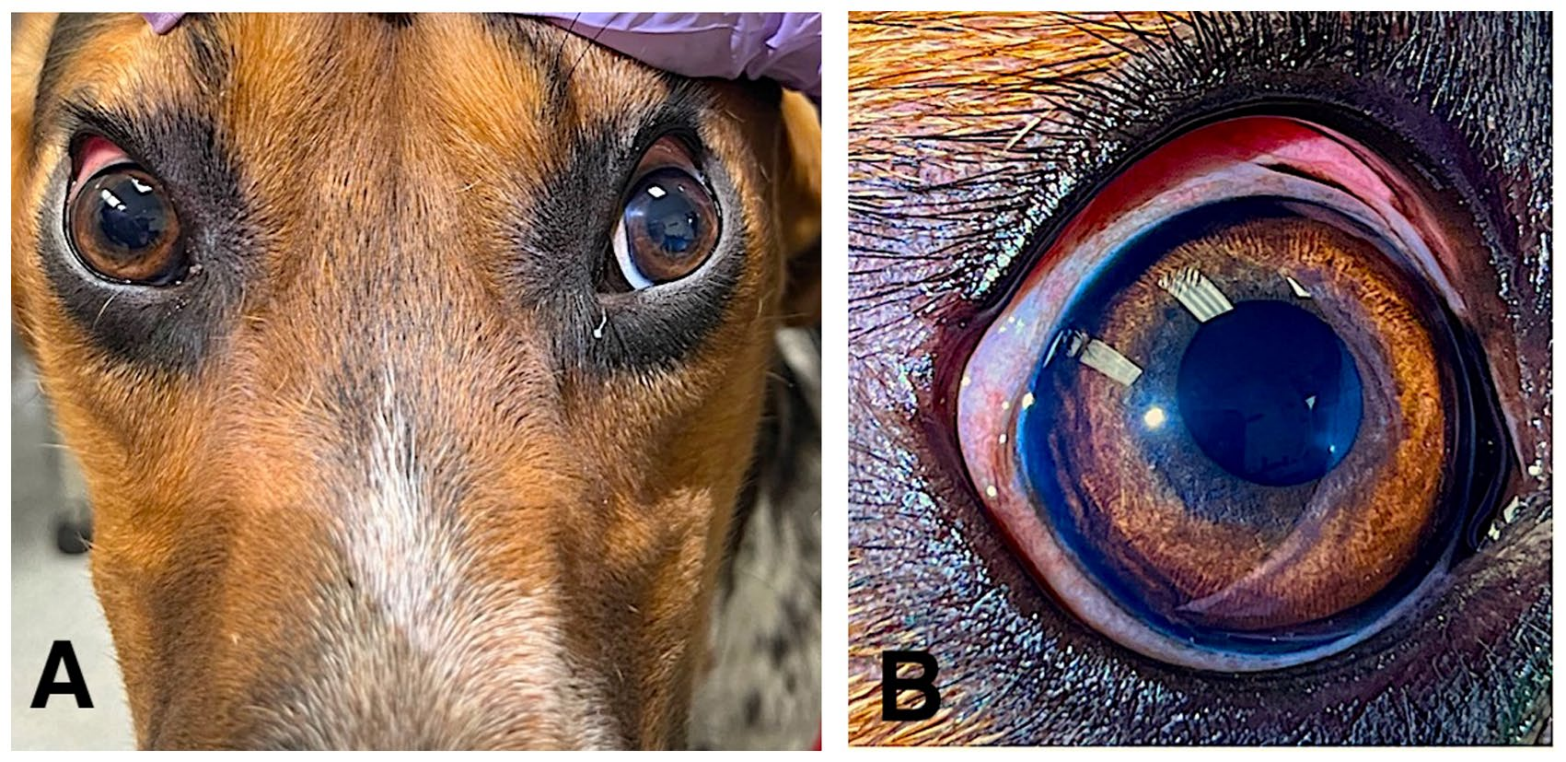
Representative images **(A,B)** of mild chemosis and hyperemia that persisted 24 hours post-PRP injection. Note that PRP injection was given in the subconjunctival space of the right eye only. The left eye received an equal volume of sterile saline.

**Figure 3:**
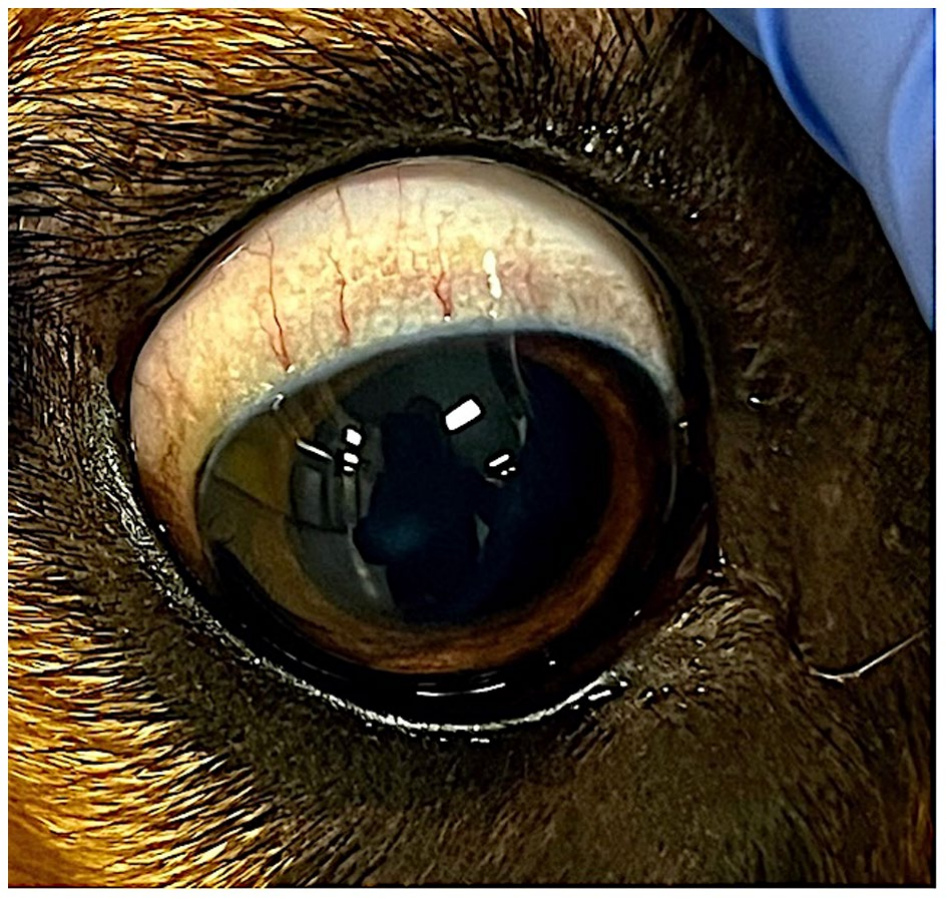
Representative image of the right eye 48 h after subconjunctival PRP injection. Note complete resolution of the chemosis and hyperemia. Three out of four dogs developed complete resolution at this time point with one dog showing complete resolution of clinical signs by 72 h post-PRP injection.

**Figure 4:**
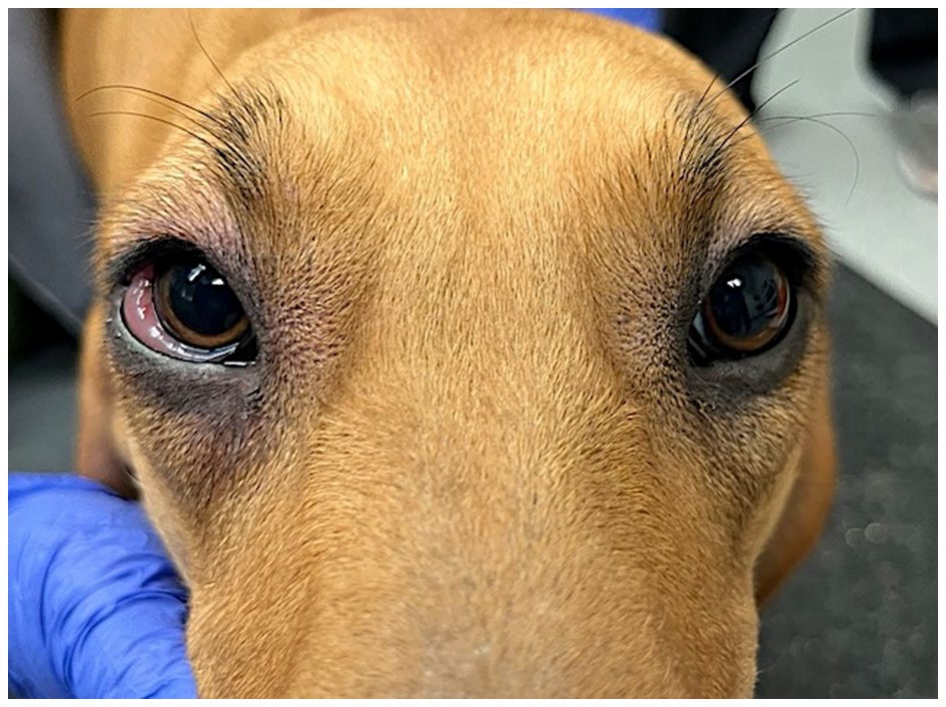
Photograph of one dog that developed periocular hyperemia one hour post-PRP injection in the subconjunctival space of the right eye. Additionally, consistent with the other dogs, is evidence of chemosis and conjunctival hyperemia of the right eye. All clinical signs, including periocular hyperemia, resolved without treatment within 72 h post-injection.

No clinically significant, serious adverse effects were observed at any time point. (0/4; exact 95% CI 0–57%) Physical examination findings and hematologic parameters remained within normal limits throughout the study period. All dogs developed a diffuse, nonpainful chemosis and conjunctival hyperemia in the PRP-treated eye within one hour of both the first and second injections (Figure 1). For all dogs (4), both conjunctival hyperemia and chemosis/conjunctival swelling were given a score of 2 according to the SPOTS criteria (35). The degree of chemosis and conjunctival hyperemia did not differ between the first and second injections. In three out of four dogs, the chemosis and hyperemia fully resolved within 48 h without any intervention (Figures 2, 3). In one dog, periocular hyperemia, most prominent nasally, was noted in addition to chemosis and conjunctival hyperemia (Figure 4). This fully resolved by 72 h post-injection without any intervention. No signs of ocular discomfort as defined by blepharospasm, discharge, pawing or rubbing at the eye or behavioral changes were noted at any time point. All other ophthalmic findings (including Schirmer tear testing, fluorescein stain testing, tonometry, slit-lamp biomicroscopy and indirect ophthalmoscopy) were normal at all time points. No adverse effects were noted at any time point in the saline-treated eye.

## Discussion

In this preliminary safety study, repeated subconjunctival administration of allogeneic, pooled freeze-dried platelet-rich plasma (PRP; VetStem PrecisePRP™ Canine) appears to be well-tolerated by normal dogs. Diffuse conjunctival hyperemia and chemosis were noted in all eyes receiving PRP injections; however, this biologic reaction to PRP appeared nonpainful with no signs of discomfort noted at any time point after injection. Additionally, conjunctival hyperemia and chemosis resolved within 48 to 72 h post-injection in all dogs without necessitation of further intervention.

Allogeneic PRP theoretically carries increased risk of undesired inflammatory or hypersensitivity reactions compared to autologous PRP. Previous studies have evaluated allogeneic PRP to classify its immunogenic potential. An *in vitro* study by Papait et al. (36) proposed that allogeneic PRP elicits a minimal immunogenic response by inducing monocyte differentiation and promoting an anti-inflammatory microenvironment. The immunogenicity of allogeneic PRP was further studied *in vivo* by Zhang et al. (16). Rabbits receiving local, intramuscular injections of allogeneic PRP did not exhibit a severe or chronic immune response, as demonstrated by assessment of peripheral blood lymphocyte populations and histological evaluation of muscle tissue at the injection site. Further studies have verified the clinical tolerability and efficacy of allogeneic PRP for its use in a variety of veterinary and human conditions such as chronic wounds and inflammatory orthopedic diseases and found no adverse reactions (37–41). The low immunogenicity of local, targeted allogeneic PRP therapy is thought to be due to a variety of factors including minimal interaction with host antibodies, degradation of PRP over several weeks leaving little opportunity for development of a chronic immune response, and possible modification of platelet antigen structure during platelet activation (37, 41).

The transient changes observed likely represent an expected, mild inflammatory response to the biologic activity of PRP, rather than an adverse pathologic reaction (42). The findings in the present study are consistent with those of previous reports investigating adverse reactions associated with allogeneic PRP therapy. Garbin et al. (18) performed a safety analysis of freeze-dried allogeneic PRP in normal equine joints and noted a mild, but significant self-limiting inflammatory response in synovial fluid defined by an increase in nucleated cell counts, prostaglandin E2, and total protein when compared to autologous PRP. This did not cause lameness or other overt adverse clinical signs. Additionally, allogeneic PRP has been utilized for intra-articular use in canine patients with no apparent adverse effects noted in both laboratory and clinical patients (21). More recently, Kooy et al. (43) compared local and systemic effects of a single intra-articular injection of equine leucoreduced allogeneic pooled freeze-dried PRP to a placebo control (saline) in normal, healthy equine joints. No differences between groups for joint swelling, joint circumference, heat scores or passive flexion were noted post-injection. Topical allogeneic PRP eye drops have also been successfully used in both human and veterinary species for the treatment of various ocular surface conditions, most importantly, dry eye disease, with no significant adverse effects noted (10, 23, 25–27).

Interestingly, autologous PRP has been shown to cause similar, mild adverse reactions to those described for allogeneic PRP. A systematic review of the current literature by Cardona-Ramirez et al. (44) evaluated 18 studies utilizing PRP for intra-articular use in canines. While there was variability in study design and PRP composition, all studies noted improvement in function and pain post-PRP intra-articular injection. Six of these studies reported adverse effects to include self-limiting local pain, inflammation and lameness that resolved 48–72 h post-injection.

Subconjunctival autologous PRP injections performed in canine patients for various ocular surface conditions have demonstrated no serious adverse effects (7, 8, 33, 34). Similar findings have been reported for subconjunctival autologous PRP injections in human patients with dry eye disease; however, a small number of cases exhibited subconjunctival hyperemia or hemorrhage, similar with the observations in this study (31, 45). Additionally, mild ocular irritation has been reported with both injection and topical application of autologous PRP for human use (31, 46).

Subconjunctival injection may be a preferred route of ocular administration in veterinary species due to a variety of factors. Topical administration is most effective when performed frequently (4–6 times daily) over at least several weeks (29, 30) which is impractical for many pet owners. Subconjunctival administration can provide more concentrated PRP delivery, which has been shown to have faster and more significant improvement in dry eye disease in humans when compared to a topical PRP formulation (31). An allogeneic PRP preparation may also provide more consistent dosing with a higher concentration of platelets and associated growth factors compared to autologous PRP. Therefore, subconjunctival administration of allogeneic PRP may, in theory, provide a better formulation and delivery method, thereby enhancing its overall efficacy for ophthalmic use. Additionally, subconjunctival injections are generally well tolerated by veterinary patients. In our study population, only minimal physical restraint and topical anesthesia were required to perform an injection. This observation is consistent with the authors’ clinical experience; however, select patients may require light sedation based on temperament.

More recently, subconjunctival injection of autologous platelet-rich fibrin (PRF) for the treatment of severe dry eye disease has been studied in humans (47). PRF may provide a more sustained benefit when compared to PRP due to the gradual release of bioactive factors through a fibrin matrix. To the authors’ knowledge, an allogeneic PRF product is not commercially available. Additionally, PRF must be injected within the first 15–20 min following a reduced centrifugation process, while it remains in its liquid state prior to polymerization. Due to these limitations, PRP was chosen over PRF for the purposes of this study and future clinical applications.

While results of this study show promise in the safety of canine subconjunctival allogeneic PRP, there are several limitations worth noting. The sample size in this study was small, and inclusion of a larger number of normal dogs may have demonstrated unanticipated, adverse outcomes not identified in the current study. Additionally, this was an open-label study, and both examiners were aware of which eye received PRP vs. saline. This may have inadvertently introduced a potential for bias into the measured outcome parameters. However, all efforts were made to use objective criteria and standardized clinical photographs when evaluating eyes. Importantly, this study evaluated reactions to subconjunctival PRP injections in normal eyes. It is possible that the minimal adverse effects seen in this study may be more pronounced in canine patients who already have evidence of ocular surface inflammatory disease causing hyperemia and irritation. Therefore, translating this data to clinical patients with underlying ocular inflammatory disease may be challenging. Further classification of the transient, inflammatory reaction noted in this study may be useful in understanding the type of reaction caused by PRP. Objective data such as conjunctival cytokine analysis could provide more insight into the type of inflammatory reaction observed post-injection and is a future consideration.

In conclusion, allogeneic PRP is safe, technically straightforward, and well-tolerated when delivered as subconjunctival injections administered 2 weeks apart in normal canine eyes. No significant adverse effects were noted other than a non-painful, transient, mild to moderate conjunctival hyperemia and chemosis. Findings from this safety study support future clinical investigation into the efficacy of allogeneic PRP for the treatment of a variety of chronic canine ocular surface inflammatory diseases, further highlighting its potential as a safe and useful regenerative therapeutic option in veterinary ophthalmology.

## Data Availability

The raw data supporting the conclusions of this article will be made available by the authors, without undue reservation.
